# Relationship between β-Carotene Accumulation and Geranylgeranyl Pyrophosphate Synthase in Different Species of *Dunaliella*

**DOI:** 10.3390/plants11010027

**Published:** 2021-12-22

**Authors:** Lu Xu, Fan Gao, Jia Feng, Junping Lv, Qi Liu, Fangru Nan, Xudong Liu, Shulian Xie

**Affiliations:** School of Life Science, Shanxi University, Taiyuan 030006, China; xl9858111@163.com (L.X.); gaofan@sxu.edu.cn (F.G.); fengj@sxu.edu.cn (J.F.); lvjunping024@sxu.edu.cn (J.L.); liuqi@sxu.edu.cn (Q.L.); nanfr@sxu.edu.cn (F.N.); liuxudong@sxu.edu.cn (X.L.)

**Keywords:** *Dunaliella*, β-carotene, geranylgeranyl pyrophosphate synthase

## Abstract

To study the relationship between β-carotene synthesis and geranylgeranyl pyrophosphate synthase (GGPS) activity, 15 species of *Dunaliella* were used to determine the changes in photosynthetic pigment contents, chlorophyll fluorescence parameters, β-carotene content, and GGPS activity. By observing the morphology and size of 15 species of *Dunaliella*, D8 has the largest individual algal cell and D9 has the smallest individual. Growth was relatively slow during days one through seven. After about eight days, the cells entered the logarithmic growth period and grew rapidly to a high density. After about 45 days, they entered a mature period, and growth slowed down. The contents of chlorophyll, carotenoids, and β-carotene increased during growth. D1 has the highest accumulation of β-carotene, and GGPS enzyme activity has a positive linear relationship with the β-carotene synthesis content. Phylogenetic analysis showed that the GGPS proteins of the 15 species were highly homologous, and the GGPS protein was not part of the membrane.

## 1. Introduction

*Dunaliella* (Chlorophyceae, Chlorophyta) [1] is a single-celled alga without cell walls [2,3]. *Dunaliella* is one of the most salt-tolerant eukaryotes found in nature. It exists in salt lakes, and 28 species have been reported. *Dunaliella salina* is often used as a model algal species [4].

*Dunaliella* has been used in food, health products, cosmetics, anti-cancer drugs, biotechnology and other fields [5,6,7,8]. The synthesis of fucoxanthin with β-carotene has great application prospects in anti-cancer, anti-inflammatory, and blood pressure reduction. In addition, other nutrients contained in the *Dunaliella* algae, such as proteins, amino acids, carbohydrates, and lipids, have great commercial development and economic value [9,10].

β-carotene plays an important role in maintaining human health. *Dunaliella* salina is one of the important raw materials for the synthesis of β-carotene [11]. Synthesis of β-carotene is jointly regulated by a variety of enzymes, including isopentenyl diphosphate (IPP), dimethylallyl pyrophosphate (DMAPP), geranylgeranyl pyrophosphate synthase (GGPS), phytoene synthase (PSY), phytoene undergoes phytoene desaturase (PDS), ζ-carotene dehydrogenation (ZDS), carotenoid isomerase (CRTISO), 15-cis-ζ-isomerase (ZISO), and lycopene β-cyclase (LCYB) [12,13,14]. Among them, GGPS is the key enzyme in β-carotene synthesis.

No study has investigated the relationship between GGPS activity and β-carotene synthesis in the *Dunaliella* algae. In this study, 15 species of *Dunaliella* were cultured, and GGPS enzyme activity and β-carotene content were determined during the logarithmic and mature phases to determine the relationship between them. The results will provide a reference for further industrial production of the *Dunaliella* algae.

## 2. Materials and Methods

### 2.1. Algae Species and Cultivation

A total of 15 *Dunaliella* species were obtained by sampling domestic saltwater lakes and purchasing from overseas algal banks. The details are shown in Table 1. *Dunaliella* medium was used to expand the *Dunaliella* algal cultures. The medium was prepared according to a previously published method, and the reagents and dosages required for use are shown in Table 2 [15]. All reagents used were analytically pure. The pH of the medium is 7.5, and the salinity is 87.69 × 10^−9^ ppt. The *Dunaliella* species were incubated in a culture room at 25 ± 2 with fluorescent lamps at a light of 55.50 μmol m^−2^ s^−1^ and a 12 h:12 h light:dark cycle. During the cultivation process, the Erlenmeyer flask needs to be shaken evenly every day.

### 2.2. Observations of Cell Morphology and Determination of Absorbance

A small amount of *Dunaliella* was fixed with Luger’s reagent and the morphology of the algal cells was observed under an Olympus BX-51 microscope (Olympus Corp, Tokyo, Japan), equipped with a DP72 digital camera.

Three mL of the algae liquid was used to measure the peak absorbance value with an ultraviolet-visible spectrophotometer (TU-1810, Persee, Auburn, CA, USA), and the result was 686 nm. The absorbance value was measured once every two days [16].

All experiments were performed three times to ensure the accuracy of the data. The values were expressed as mean ± SD of three parallel measurements. A one-way analysis of variance (ANOVA) in SPSS software was used to evaluate the significance of the differences between algae. *p* < 0.05 (*) indicated the presence of a statistically significant difference, *p* < 0.01 (**) was considered highly significant, and *p* > 0.05 meant no significant difference.

### 2.3. Determination of Photosynthetic Pigment Content and the Chlorophyll Fluorescence Parameters

The determination of the chlorophyll content is based on Mera’s method with some modifications [17]: Take 6 mL of algal liquid in a 10 mL centrifuge tube, centrifuge at 4500× *g* for 5 min, pour out the supernatant, add 5 mL of 95% ethanol, mix well, put it in a refrigerator at 4 °C for 24 h, take it out for 8000 × *g* of centrifuge for 10 min, filter the supernatant with a 0.45 μm filter, put 3 mL into the cuvette of the UV-Vis spectrophotometer, and measure the OD values at 649, 665, and 470 nm wavelengths; calculate the content of Chlorophyll *a* (Chl *a*), Chlorophyll *b* (Chl *b*), and Carotenoid (Car) according to the following formulas, all in mg/L.

(1) Chlorophyll a (mg/mL)=13.95× OD (665 nm)−668× OD (649 nm)

(2) Chlorophyll b (mg/mL)=24.96× OD (649 nm)−7.32× OD (665 nm)

(3) $$\mathrm{Carotenoids}\;\left(\mathrm{mg}/\mathrm{mL}\right)=\frac{\left(1000\times \mathrm{OD}\left(470\;\mathrm{nm}\right)-2.05\times \mathrm{Chl}\;a-114.8\times \mathrm{Chl}\;b\right)}{245}$$

The chlorophyll fluorescence parameters were measured using a previous method [18]: Put 3 mL of the *Dunaliella* sample solution in a centrifuge tube, then wrap it in tin foil, and treat it in the dark for 30 min; use a portable PAM chlorophyll fluorometer (AquaPen-CAP-C 100, Ecotech, Shanghai, China) to sequentially determine the Fv/Fm and Fv/Fo ratios of each algal solution, and use the professional FluorPen software for data transmission and analysis.

### 2.4. Determination of β-Carotene Content

The β-carotene standard curve was established following a previous method [19]. Two liters of a mixture of acetone and petroleum ether were prepared at a ratio of 3:1.

Six mL of algal liquid was crushed with an ultrasonic crusher (SCIENTZ-IID, Scientz, Ningbo, China); after extracting β-carotene with acetone-petroleum ether mixture, the β-carotene content was determined by spectrophotometry [20]. The absorbance was measured at 452 nm after the liquid was concentrated. β-carotene content was determined according to the Biaoqu formula.

### 2.5. Determination of the GGPS Activity

Forty-five mL of algal liquid was repeatedly frozen and thawed after crushing with an ultrasonic crusher, using a kit (ELISA, FanKew, Shanghai, China) to measure the GGPS activity. A microplate reader (Epoch2, BioTek, Winooski, VT, USA) was used to measure the absorbance at 450 nm, and the Biaoqu formula was used to calculate the GGPS activity.

### 2.6. Amplification, Sequencing, and Characterization of GGPS in Dunaliella

The TaKaRa MiniBEST Universal RNA Extraction Kit (Takara, Shiga, Japan) was used for the extraction of RNA from *Dunaliella*. The PrimeScript™ RT reagent Kit with gDNA Eraser (Takara, Shiga, Japan) was used for reverse transcription. The cDNA sequences for GGPS genes in *Dunalella saline* which have been published in the NCBI were downloaded and divided into three cDNA sequence fragments with repeating areas. Using them as a template to design primers for the NCBI, three primers were obtained, and the results are shown in Table 3; the primers required for the three-stage nested PCR were synthesized by Sangon Biotech (Shanghai, China). Using them to amplify the cDNA in *Dunaliella* and then using the SEQUENTER software to splice between three sequences, the splicing sites are at 534 ± 11 bp and 800 ± 10 bp. Finally, the length of the GGPS gene in the 15 *Dunaliella* species is 1715 ± 15 bp.

The PCR reaction system was 20 μL, including 2 μL of template cDNA, 2 μL of dNTP, 2 μL of DNA buffer, 0.2 μL of Easy Taq enzyme, 2 μL of upstream primer, 2 μL of downstream primer, and 9.8 μL of ultrapure water.

The PCR amplification program was 94 °C pre-denaturation for 3 min, 94 °C denaturation for 1 min, Tm′ annealing for 1 min, 72 °C of extension for 1.30 min and 40 cycles, and a 72 °C extension for 10 min. The products were stored at 4 °C.

The amplified products were electrophoresed (DYY-7C, LIUYI, Shanghai, China) on a 1% agarose gel. After sequencing, the Sequencher software was used to (https://www.sequencher.cn/ (accessed on 23 November 2021)) splice the sequence and obtain the GGPS nucleotide sequence.

MEGA 7 and Clustal W software were used to analyze the genetic distance of the GGPS genes from the 15 *Dunaliella* species [21,22,23]. The phylogenetic tree of the *Dunaliella* GGPS gene was constructed using the neighbor-joining method [24]. The online tool, TMHMM-2.0, and a protein domain database (SMART database: http://smart.embl-heidelberg.de/ (accessed on 23 November 2021)) were used to analyze the protein transmembrane situation of the GGPS between different species.

## 3. Results

### 3.1. Dunaliella Growth and Cell Morphology

**Figure 1:**
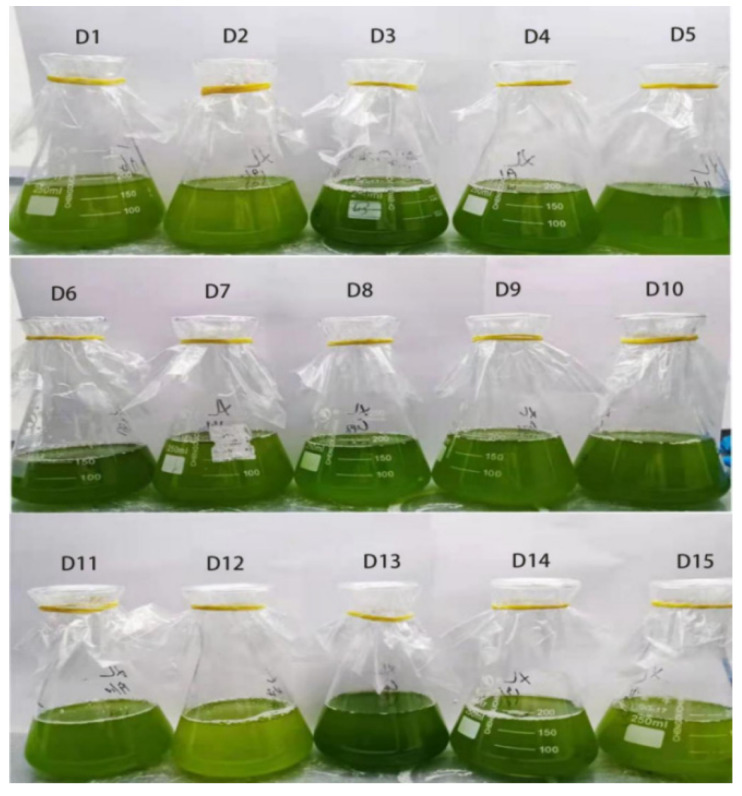
Appearance of the 15 *Dunaliella* species in Erlenmeyer flasks after 40 days of culture.

**Figure 2:**
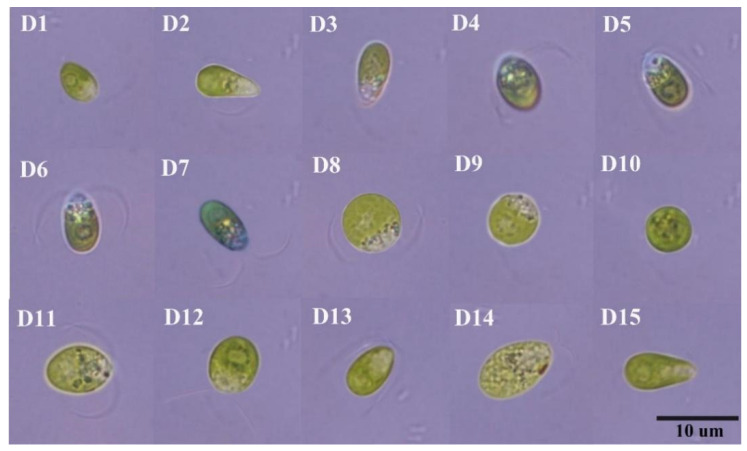
Algal cell morphology of the 15 species of *Dunaliella* (100×).

Figure 1 shows the state of the algae liquid when the 15 species of *Dunaliella* were cultured in an Erlenmeyer flask for 40 days, and Figure 2 and Table 4 show the microscopic morphology of the algal cells after 40 days of culture. The results showed that the color of the algae cells of 15 species of *Dunaliella* was green. The algae cells of D2, D5, and D15 are pear-shaped, D8, D9, and D10 are round, and the others are oval. The algal cells of D2, D4, D5, D6, D7, D8, D11, D12, D13, and D14 have flagella. Among the 15 *Dunaliella* species, D8 has the largest individual cell with a length and width of 10.25 ± 2 μm, and D9 has the smallest individual cell with a length and width of 6.9 ± 0.5 μm and 4.6 ± 0.2 μm, respectively. For the remaining 13 strains of *Dunaliella*, the overall width range of the algae cells is 3.8–8.4 μm, and the height range is 6.5–10.3 μm. For the cell height, with width as a single factor, a one-way analysis of variance was performed, and pairwise comparisons were made. The results are shown in Table 4. When the letter marks are the same, it means that there is no significant difference between them. According to the markers in Table 4, D8 is marked as B, D1 and D15 are marked as A, and the other 12 *Dunaliella* strains are marked with AB. It means that there is a significant difference between D8 and D1, D15, *p* < 0.05. The 15 strains of *Dunaliella* have no obvious differences as a whole.

**Figure 3:**
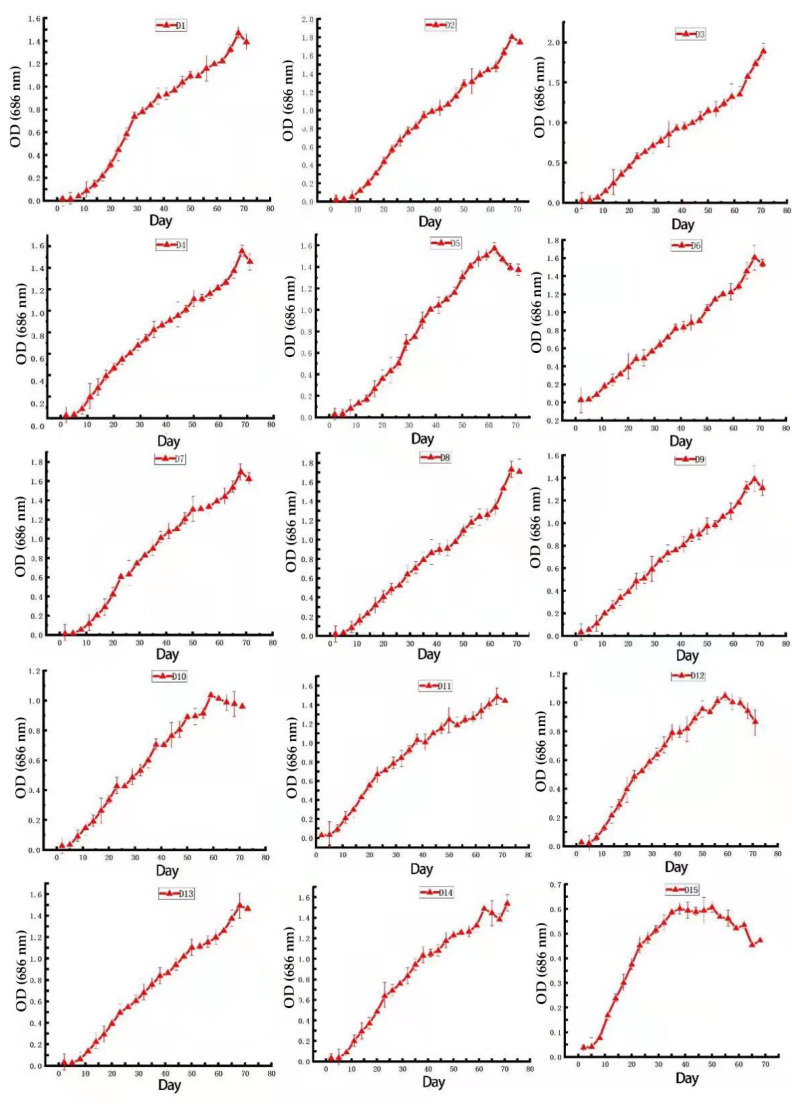
Growth changes in the 15 *Dunaliella* species.

Figure 3 shows that a small amount of algae was inoculated into a tapered bottle containing a *Dunaliella* medium, and the growth and reproduction of the algal cells was slow at days one to seven. The algal cells grew rapidly, and their density was high beginning from the 8th day into the logarithmic growth phase. After about 45 days, the algal species entered the mature stage, and the growth rate slowed. Among the 15 *Dunaliella* species, D3 grew the worst, and D15 grew the best.

### 3.2. Comparison of Photosynthetic Pigment Contents and Chlorophyll Fluorescence Parameters

Supplementary Figures S1–S3, respectively, show the changes in chlorophyll *a*, chlorophyll *b*, and carotenoid contents in the 15 *Dunaliella* species. All three pigments increased over time. Among them, the chlorophyll *a*, chlorophyll *b*, and carotenoids of D8 are the largest (chlorophyll *a* content is 19.58 mg/mL, chlorophyll *b* content is 10.3 mg/mL, carotenoid content is 6.4 mg/mL). Compared with 14 other *Dunaliella* strains, D15 has the lowest chlorophyll and carotenoid content (chlorophyll *a* content is 7.30 mg/mL, and the carotenoid content is 1.881 mg/mL). It shows that the chlorophyll and carotenoid synthesis in different *Dunaliella* varies, but the overall change trend has gradually accumulated over time. D8 can be used as a selected algae species of chlorophyll and carotenoids in *Dunaliella.*

The Fv/Fo and Fv/Fm values of the 15 *Dunaliella* species changed similarly, they all increased first and then decreased (Supplementary Figures S4 and S5). The D2, D6, D8, and D14 species reached the maximum on day eight; the D1, D7, D9, D10, and D13 species reached the maximum on day 11, and the D3, D5, D6, D11, D12, and D15 species reach the maximum on day 15. It shows that the chlorophyll fluorescence parameters in different *Dunaliella* have differentiation, but the overall change trend is the same, and they are decreasing first.

### 3.3. Comparison of β-Carotene Content

Using β-carotene powder as the standard, the regression equation for the standard curve was (correlation coefficient = 0.9945):

(4) Y=0.2687x−0.0051

**Figure 4:**
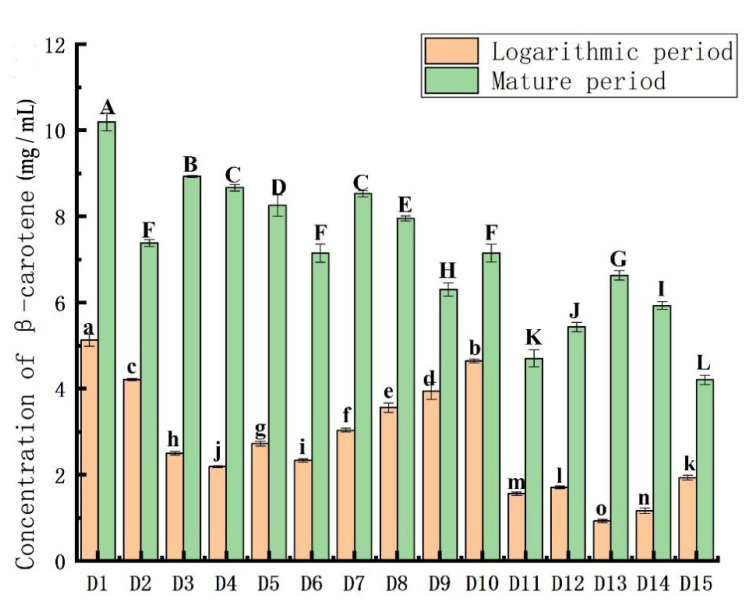
Comparison of β-carotene content in the 15 *Dunaliella* strains. The letters a–i and A–L in the figure represent the single-factor significant difference analysis of the β-carotene content in 15 *Dunaliella* strains at the logarithmic and mature stages, respectively. When the letters marked on different *Dunaliella* species are the same, it means that there is no significant difference between them, and when the letters are different, there is a significant difference.

Figure 4 shows that the β-carotene content of *Dunaliella* at the mature stage was significantly higher than that during the logarithmic stage, indicating that the β-carotene content accumulated over time. The D1 strain had the highest β-carotene content of 10.19 × 10^−2^ mg/mL, and D15 had the lowest β-carotene content of 4.3 × 10^−2^ mg/mL.

The specific comparison during the logarithmic phase was: D1 > D10 > D2 > D9 > D8 > D7 > D5 > D3 > D6 > D4 > D15 > D12 > D11 > D14 > D13. In the logarithmic phase, the one-way analysis of variance for β-carotene is indicated by lowercase letters a–o. The results showed that the β-carotene content of 15 *Dunaliella* species in the logarithmic phase were all significantly different, *p* < 0.05.

The specific comparison during the mature phase was: D1 > D3 > D1 > D4 > D7 > D8 > D10 > D2 > D6 > D13 > D9 > D14 > D12 > D11 > D15. In the mature period, the single-factor significant difference analysis of β-carotene is indicated by capital letters A–L. The results showed that D2, D6, and D10 had no significant difference, D4 and D7 had no significant difference, *p* > 0.05, and the other 10 *Dunaliella* species had significant differences, *p* < 0.05.

### 3.4. Comparison of GGPS Activity

**Figure 5:**
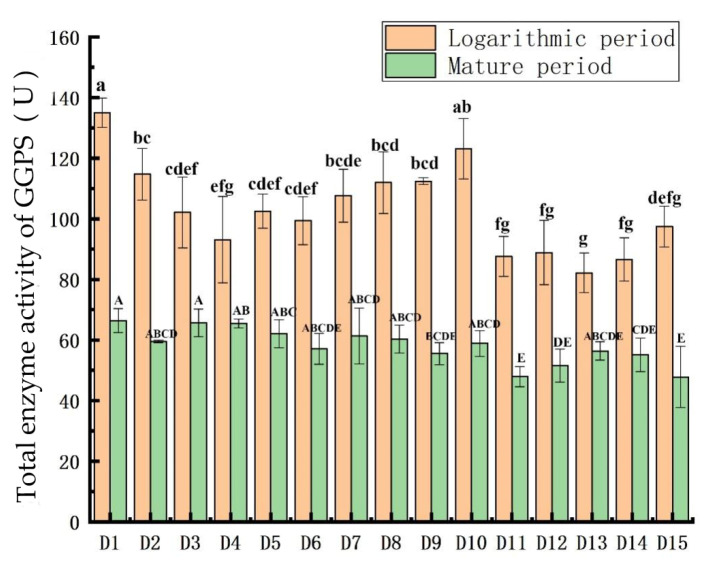
Comparison of GGPS activity in the 15 *Dunaliella* strains. The letters a–g and A–E in the figure represent the single-factor significant difference analysis of the GGPS total enzyme activity content of 15 *Dunaliella* strains in the logarithmic and mature stages, respectively. When the letters marked on different *Dunaliella* species are the same, it means that there is no significant difference between them, and when the letters are different, it means that there is a significant difference.

Figure 5 shows that the total GGPS activity was significantly lower during the mature phase than during the logarithmic phase.

The specific comparison during the logarithmic phase was: D1 > D10 > D2 > D9 > D8 > D7 > D5 > D3 > D6 > D15 > D4 > D12 > D11 > D14 > D13. In the logarithmic phase, the one-way analysis of variance of GGPS enzyme activity is represented by lowercase letters a–g. The results show that, except for D10, D1 is significantly different from the other 13 algae; except for D2, D7, D8 and D9, D10 is significantly different from the other 10 algae; except for D11, D12, D14 and D15, D13 has significant differences from the other 10 species of algae (*p* < 0.05).

The specific comparison during the mature phase was: D1 > D3 > D1 > D4 > D7 > D8 > D2 > D10 > D6 > D13 > D9 > D14 > D12 > D11 > D15. In the mature period, the one-way analysis of variance of the GGPS enzyme activity is represented by capital letters A–E. The result shows that D1 is significantly different from D9, D11, D14, and D15; D11 is significantly different from D2, D3, D4, D5, D7, D8, and D10 (*p* < 0.05).

### 3.5. Relationship between β-Carotene Content and GGPS Activity

**Figure 6:**
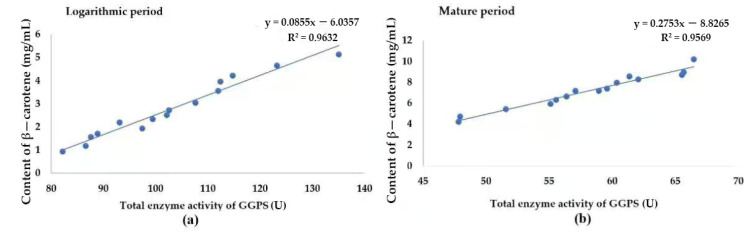
Linear relationship of β-carotene and GGPS enzyme activity in two periods. (**a**) In the logarithmic period. (**b**) In the mature period.

Figure 6 shows the linear relationship between β-carotene and the GGPS enzyme activity. In the logarithmic period, the formula for the linear relationship between the two is: y=0.0855x−6.0357, and the coefficient is 0.9632. In the mature period, the formula for the linear relationship between the two is: y=0.2753x−8.8265, and the coefficient is 0.9569. It can be seen that the relationship between the two is linear and positive.

### 3.6. Amplification and Characterization of the GGPS Gene

**Figure 7:**
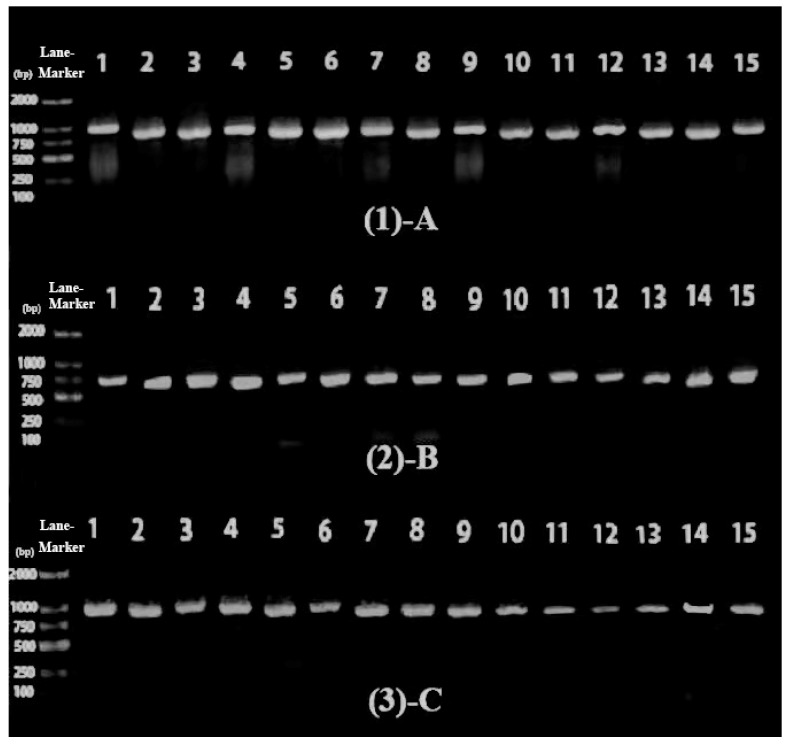
Lanes (1), (2), and (3) represent the cDNA amplification products, respectively, obtained by amplification with primers A, B, and C; from left to right: the D1, D2, D3, D4, D5, D6, D7, D8, D9, D10, D11, D12, D13, D14, and D15 species.

Figure 7 shows the cDNA sequence amplification results obtained after electrophoresis of the 15 *Dunaliella* species using PCR with three sets of different primers. The electrophoresis results are compared with Lane-Marker as the control standard, and the leftmost bp indicates the length of the strip. As can be seen, the amplification result is good, as all were positive and there were no contaminated bands. The amplified sequence length of primers A and C was about 1000 bp and that of primer B was about 750 bp.

Table 5 shows the genetic distances of the GGPS genes among the 15 *Dunaliella* species. Among the 15 *Dunaliella* species, D8 and D13 were the same species of *Dunaliella*, and the genetic difference and genetic distances between them were zero; D5 and D12 were the same species of *Dunaliella*, and the genetic difference between them was 0.01, with a genetic distance of 14 bp. The difference in the GGPS genes between the *Dunaliella* species was 0–3%, and length was 0–43 bp. The GGPS genetic distances of the same *Dunaliella* species were quite different.

**Figure 8:**
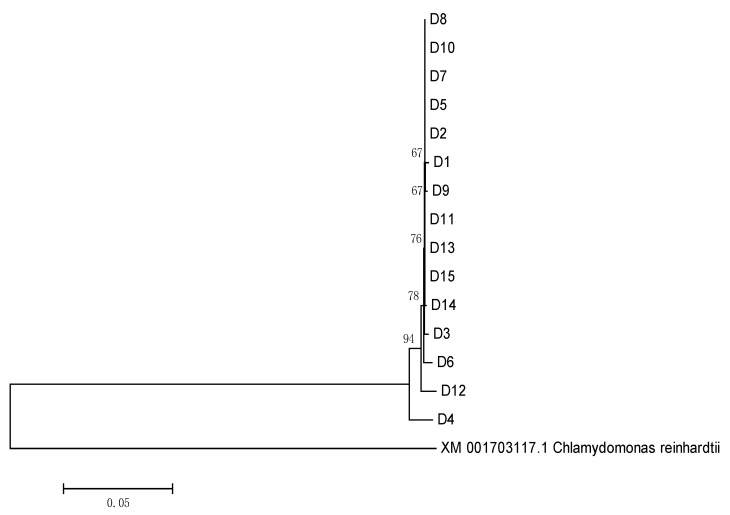
GGPS phylogenetic tree of the 15 *Dunaliella* species based on the neighbor-joining algorithm.

The GGPS genes of the 15 species of *Dunaliella* were phylogenetically analyzed by constructing a neighbor-joining tree, and the GGPS gene of *Chlamydomonas reinhardtii* was selected as the outgroup. The results are shown in Figure 8. As a result, the GGPS genes of the 15 species of *Dunaliella* were classified as one big branch. The large branch was divided into two smaller branches. The D4 strain formed a small branch alone, which was relatively but distantly related to the other 14 *Dunaliella* species.

Combining the phylogenetic tree with the above-mentioned genetic distance analysis showed that the GGPS genes have high homology, but the genetic distance between the same species was relatively high, and the establishment support rate was low, which was not suitable for classifying the same species.

In addition, the analysis of the protein transmembrane domain of the GGPS gene in the 15 species of *Dunaliella* species showed that GGPS had no protein transmembrane domain.

## 4. Discussion

Fifteen species of *Dunaliella* were cultured with the *Dunaliella* medium, and the algae cells of *Dunaliella* without cell walls were observed under a microscope; the shapes were mainly oval, pear-shaped, or round. The 15 species of *Dunaliella* are different in morphology, size, and growth cycle. The reason for the difference lies not only in the difference in algae, but also in the growth rate and suitable salinity of each algae strain [25,26]. Among the 15 *Dunaliella* species, D8 has the largest individual cell. This result is consistent with the results of Borowitzk’s research [27].

Chlorophyll fluorescence parameters are important indicators for evaluating microalgae growth, photosynthesis, and cold and heat tolerance [28,29]. The changes in the ratio of Fv/Fm and Fv/Fo during the growth process reflect the changes in the photosynthetic activity of the *Dunaliella* species; the trends all increase first and then decrease. Oukarroum studied the changes of the chlorophyll fluorescence parameters during the growth of Chlorella [30], which result in the same as this article.

The photosynthetic pigments and β-carotene content accumulated with time. The D1 strain (*D. primolecta*) had the highest β-carotene content, and D8 (*D. salina*) had the highest photosynthetic pigment contents, suggesting its use in industrial production. Liu [31] compared the β-carotene content of ten *Dunaliella* species, and the results showed that *D. salina* was the highest and *D. parva* was the lowest, which was different from the results determined here. The reason for the difference may be due to different habitats, different culture conditions, or different β-carotene extraction methods. It is also possible that the algal species changed during long-term culture.

GGPS catalyzes the formation of GGPP from IPP and DMAPP. GGPP forms β-carotene under the catalysis of PSY, PDS, ZDS, CRTISO, ZISO, and LCYB. Sun’s research on methods to increase the production of β-carotene showed that the addition of GGPS can increase the production of β-carotene [32]. The result is consistent with the positive correlation between β-carotene and the GGPS enzyme activity in this paper.

Preetha used ITS and 18S rDNA to study the phenotype and genetic diversity of different *Dunaliella* species from Indian salt marshes [33], and the results showed that *D.viridis* and *D. salina*, *D. bioculata*, *D. parva*, *D. primolecta*, and *D. tertiolecta* are relatively distantly related, and the same species of *Dunaliella* converge into a branch. In this paper, the phylogenetic analysis of the GGPS gene in *Dunaliella* shows that D4 (*D. viridis*) has a relatively distant relationship with the other 14 *Dunaliella* species. D8 and D13 are both *D. salina*, and D5 and D12 are both *D. bioculata*, but the same species do not form independent branches. Combining the above comparison and the analysis of genetic distance, it can be seen that GGPS is not suitable as a reference gene for the classification of *Dunaliella* species.

## 5. Conclusions

Fifteen species of *Dunaliella* were used to determine the changes in the photosynthetic pigment content, chlorophyll fluorescence parameters, β-carotene content, and GGPS activity. The results showed that *Dunaliella* existed in the form of a single oval, pear-shaped, or round, light green cells without a cell wall, but there were some differences in the sizes of the different species, D8 has the largest individual algae cell, and D9 has the smallest individual. Growth was relatively slow during days one through seven, a growth stagnation period. About eight days after entering the logarithmic growth phase, the cells grew rapidly to a high density. After about 45 days, they entered the mature period, and their growth slowed. The contents of chlorophyll, carotenoids, and β-carotene increased over time. The accumulation of β-carotene mainly occurred during the mature period. The β-carotene content of D1 was the highest. The species with high GGPS activity also had high β-carotene content. The phylogenetic analysis showed that the GGPS proteins of the 15 species were highly homologous, and there were no transmembrane proteins.

## Figures and Tables

**Table 1:** Algae information for the 15 *Dunaliella* species.

No.	Strain	Source
D1	*D. primolecta*	Culture Collection of Algae and Protozoa (CCAP 11/34)
D2	*D. maritima*	Culture Collection of Algae and Protozoa (CCAP 19/1)
D3	*D. peircei*	Culture Collection of Algae and Protozoa (CCAP 19/2)
D4	*D. viridis*	Culture Collection of Algae and Protozoa (CCAP 19/3)
D5	*D. bioculata*	Culture Collection of Algae and Protozoa (CCAP 19/4)
D6	*D. quartolecta*	Culture Collection of Algae and Protozoa (CCAP 19/8)
D7	*D. parva*	Culture Collection of Algae and Protozoa (CCAP 19/10)
D8	*D. salina*	Culture Collection of Algae and Protozoa (CCAP 19/12)
D9	*D. polymorpha*	Culture Collection of Algae and Protozoa (CCAP 19/14)
D10	*D. tertiolecta*	Culture Collection of Algae and Protozoa (CCAP 19/22)
D11	*D. acidophila*	Culture Collection of Algae and Protozoa (CCAP 19/35)
D12	*D. bioculata*_C33	Culture Collection of Algae at the University of Texas at Austin (LB199)
D13	*D. salina*	Salt Lake in Yuncheng City, Shanxi Province
D14	*Dunaliella* sp.	Institute of Hydrobiology, Chinese Academy of Sciences
D15	*D. apiculata*	National Center for Marine Algae and Microbiota (UW 481)

**Table 2:** *Dunaliella* medium.

Formulate Content	Components	Dosage
*Dunaliella* medium	NaCl	87.69 g
	NaNO_3_	0.42 g
	NaH_2_PO_4_·2H_2_O	0.0156 g
	CaCl_2_·2H_2_O	0.044 g
	KCl	0.074 g
	MgSO_4_·7H_2_O	1.23 g
	NaHCO_3_	0.84 g
	FeC_6_H_5_O_7_	0.002 g
	UPW	1000 mL
A5	H_3_BO_3_	286 mg
	MnCl_2_·4H_2_O	181 mg
	ZnSO_4_·7H_2_O	22.2 mg
	Na_2_MoO_4_·2H_2_O	39 mg
	CuSO_4_·5H_2_O	7.9 mg
	Co (NO_3_)_2_·6H_2_O	4.9 mg
	UPW	100 mL

**Table 3:** The primer names are A, B, and C, respectively. Tm′ is the representative annealing temperature.

Primer	Forward Primer	Reverse Primer	Tm′
A	5′-AAGAGCGAAAACGAGGAGGT-3′	5′-ATTCAGGGCGCTTCAAGGTT-3′	56 °C
B	5′-GGTGCACACCATGAGCCT-3′	5′-TGGCAGGGCACTGCTCTTA-3′	56 °C
C	5′-AGGGGGTTGTAGACCGTGTT-3′	5′-CTCCACTGTCACGTCGTCCG-3′	54 °C

**Table 4:** Description of the cell characteristics of the 15 *Dunaliella* species. The length/width of the cell is used as a single factor for single-factor analysis of variance, and the results are represented by A and B.

Species	No.	Height (H)	Width (W)	Shape	Anova (H/W)
*D. primolecta*	D1	7.7 ± 0.8 μm	4 ± 0.2 μm	Oval	A
*D. maritima*	D2	8.3 ± 1.2 μm	6.3 ± 0.9 μm	Pear	AB
*D. peircei*	D3	7.3 ± 0.6 μm	7.3 ± 0.5 μm	Oval	AB
*D. viridis*	D4	9 ± 1.3 μm	7 ± 1 μm	Oval	AB
*D. bioculata*	D5	8.7 ± 1.1 μm	4.7 ± 0.3 μm	Pear	AB
*D. quartolecta*	D6	9 ± 1.3 μm	7 ± 1.2 μm	Oval	AB
*D. parva*	D7	9.3 ± 1.2 μm	6.3 ± 1.1 μm	Oval	AB
*D. salina*	D8	10.3 ± 2 μm	10.3 ± 1.9 μm	Round	B
*D. polymorpha*	D9	6.9 ± 0.5 μm	4.6 ± 0.2 μm	Round	AB
*D. tertiolecta*	D10	7.3 ± 0.8 μm	6.7 ± 0.8 μm	Round	AB
*D. acidophila*	D11	9.3 ± 1.1 μm	6.9 ± 0.5 μm	Oval	AB
*D. bioculata*_C33	D12	8.7 ± 1 μm	6.7 ± 0.8 μm	Oval	AB
*D. salina*_42	D13	9.2 ± 1.5 μm	7.2 ± 1.2 μm	Oval	AB
*Dunaliella* sp.	D14	8.7 ± 1 μm	6.8 ± 1 μm	Oval	AB
*D. apiculata*	D15	9.7 ± 1.4 μm	6.7 ± 0.7 μm	Pear	A

**Table 5:** GGPS genetic distance among 15 *Dunaliella* species.

	D8	D10	D7	D5	D2	D1	D9	D11	D13	D15	D14	D3	D6	D12	D4	*Chlamydomonas*
D8		0	0	0	0	3	2	0	0	0	1	3	7	14	29	635
D10	0.00		0	0	0	3	2	0	0	0	1	3	7	14	29	635
D7	0.00	0.00		0	0	3	2	0	0	0	1	3	7	14	29	635
D5	0.00	0.00	0.00		0	3	2	0	0	0	1	3	7	14	29	635
D2	0.00	0.00	0.00	0.00		3	2	0	0	0	1	3	7	14	29	635
D1	0.00	0.00	0.00	0.00	0.00		4	3	3	3	4	6	10	17	32	638
D9	0.00	0.00	0.00	0.00	0.00	0.00		2	2	2	3	5	9	16	31	637
D11	0.00	0.00	0.00	0.00	0.00	0.00	0.00		0	0	1	3	7	14	29	635
D13	0.00	0.00	0.00	0.00	0.00	0.00	0.00	0.00		0	1	3	7	14	29	635
D15	0.00	0.00	0.00	0.00	0.00	0.00	0.00	0.00	0.00		1	3	7	14	29	635
D14	0.00	0.00	0.00	0.00	0.00	0.00	0.00	0.00	0.00	0.00		4	8	15	30	635
D3	0.00	0.00	0.00	0.00	0.00	0.00	0.00	0.00	0.00	0.00	0.00		10	17	30	637
D6	0.00	0.00	0.00	0.00	0.00	0.01	0.01	0.00	0.00	0.00	0.00	0.01		21	32	639
D12	0.01	0.01	0.01	0.01	0.01	0.01	0.01	0.01	0.01	0.01	0.01	0.01	0.01		43	637
D4	0.02	0.02	0.02	0.02	0.02	0.02	0.02	0.02	0.02	0.02	0.02	0.02	0.02	0.03		640
*Chlamydomonas*	0.39	0.39	0.39	0.39	0.39	0.39	0.39	0.39	0.39	0.39	0.39	0.39	0.39	0.39	0.39	

## Data Availability

Data available on request from the corresponding author.

## Supplementary Materials

The following are available online at https://www.mdpi.com/article/10.3390/plants11010027/s1, Figure S1: Changes in chlorophyll *a* content in the 15 *Dunaliella* species, Figure S2: Changes in chlorophyll *b* content in the 15 *Dunaliella* species, Figure S3: Changes in carotenoid content in the 15 *Dunaliella* species, Figure S4: Changes in the Fv/Fo ratio in the 15 *Dunaliella* species, Figure S5: Changes in the Fv/Fm ratio in the 15 *Dunaliella* species.
