# Supplementary material for: Relationship between β-Carotene Accumulation and Geranylgeranyl Pyrophosphate Synthase in Different Species of Dunaliella
Source: Plants (Basel). 2021 Dec 22;11(1):27. doi: 10.3390/plants11010027 (PMC8747272; doi:10.3390/plants11010027)
Supplement: Supplementary file 1 [file plants-11-00027-s001.zip › plants-1512631-supplementary.pdf]

Relationship between β -carotene accumulation and Geranylgeranyl Pyrophosphate Synthase in Different Species of *Dunaliella*

XU Lu[1], Fan Gao[1], FENG Jia[1], LV Junping[1], LIU Qi[1], Fangru Nan[1], LIU Xudong[1], XIE Shulian[1,*]

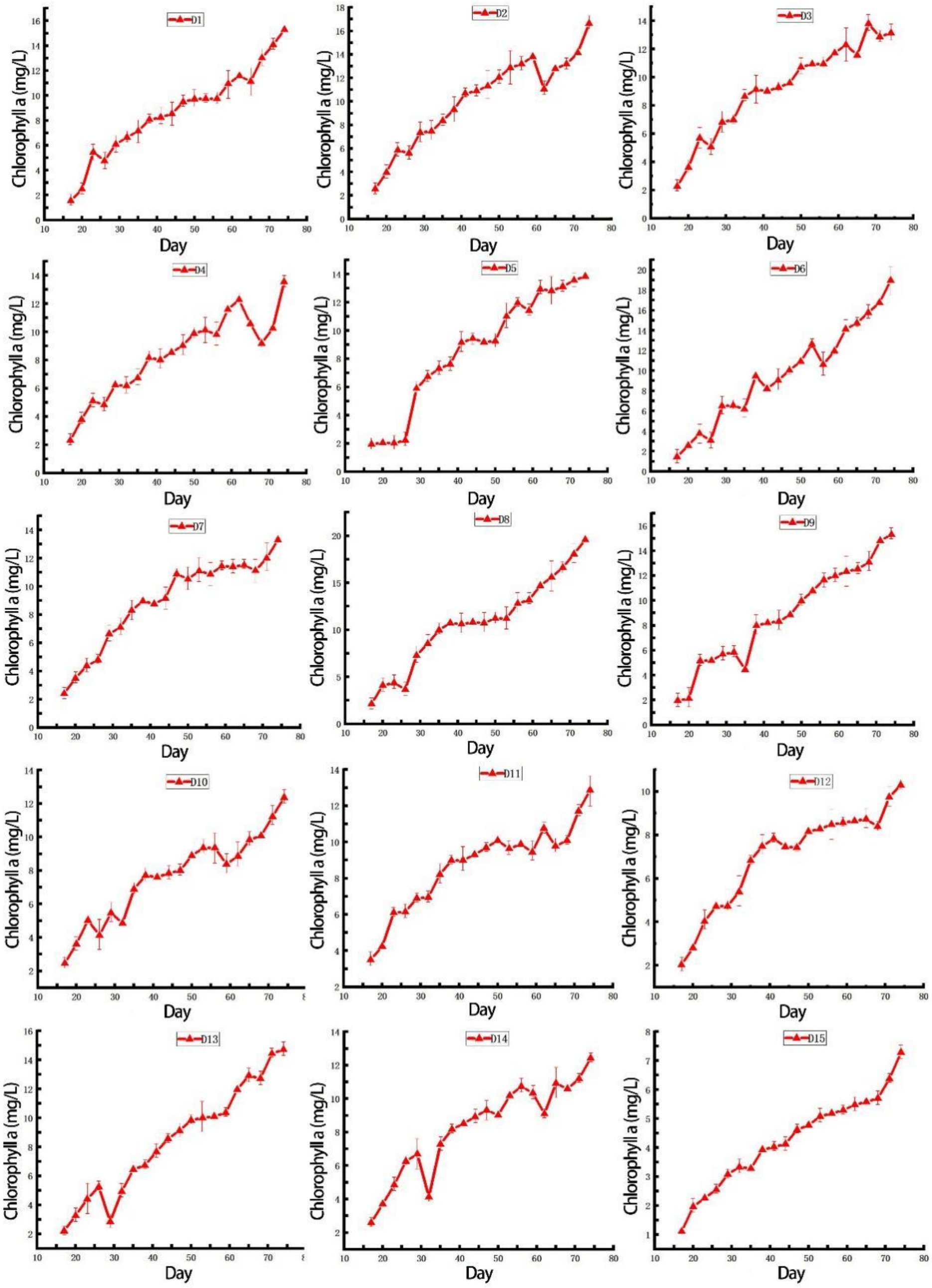

Supplementary Figure S1 Changes in chlorophyll *a* content in the 15 *Dunaliella* species

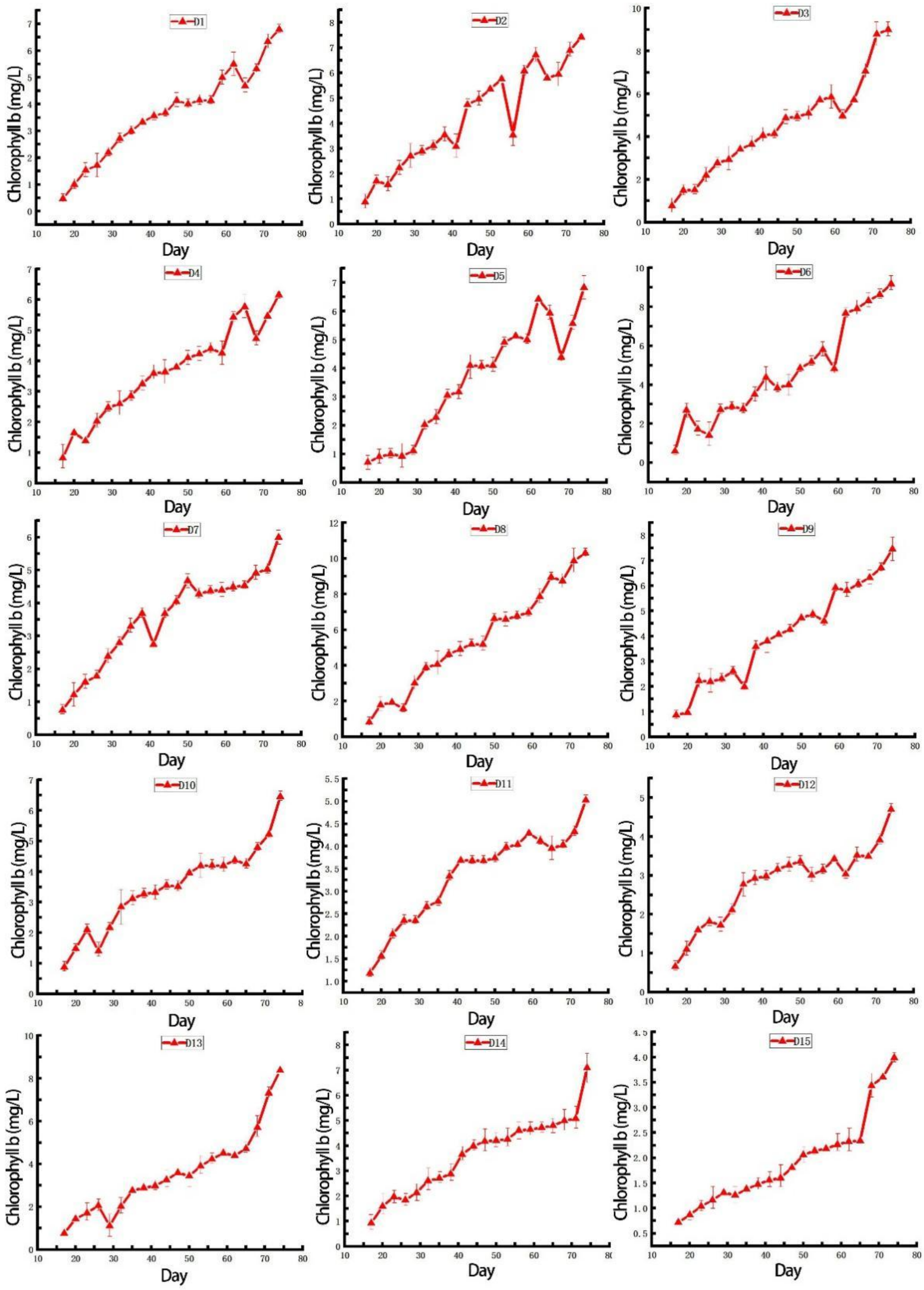

Supplementary Figure S2 Changes in chlorophyll *b* content in the 15 *Dunaliella* species

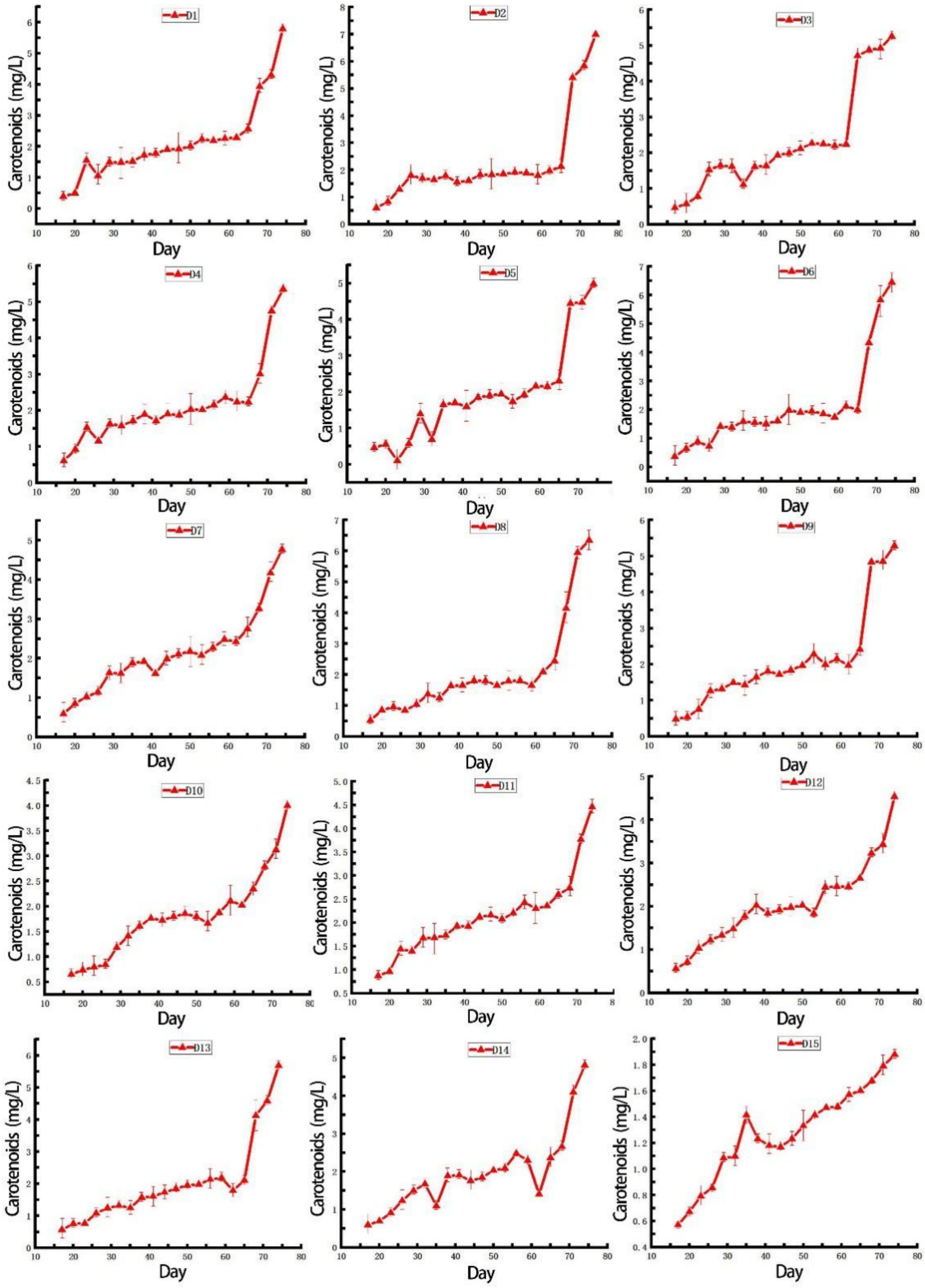

Supplementary Figure S3 Changes in carotenoid content in the 15 *Dunaliella* species

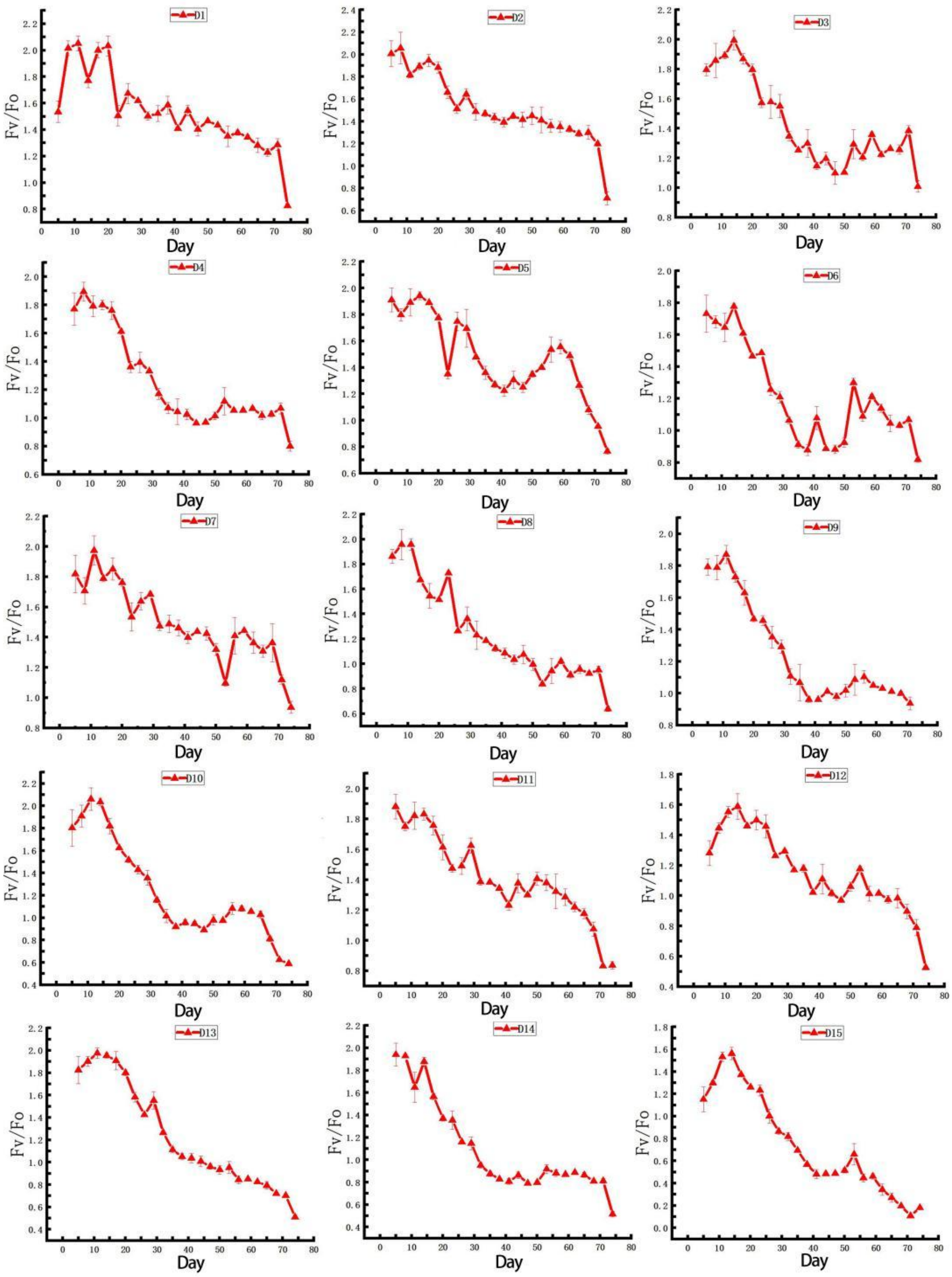

Supplementary Figure S4 Changes in the Fv/Fo ratio in the 15 *Dunaliella* species

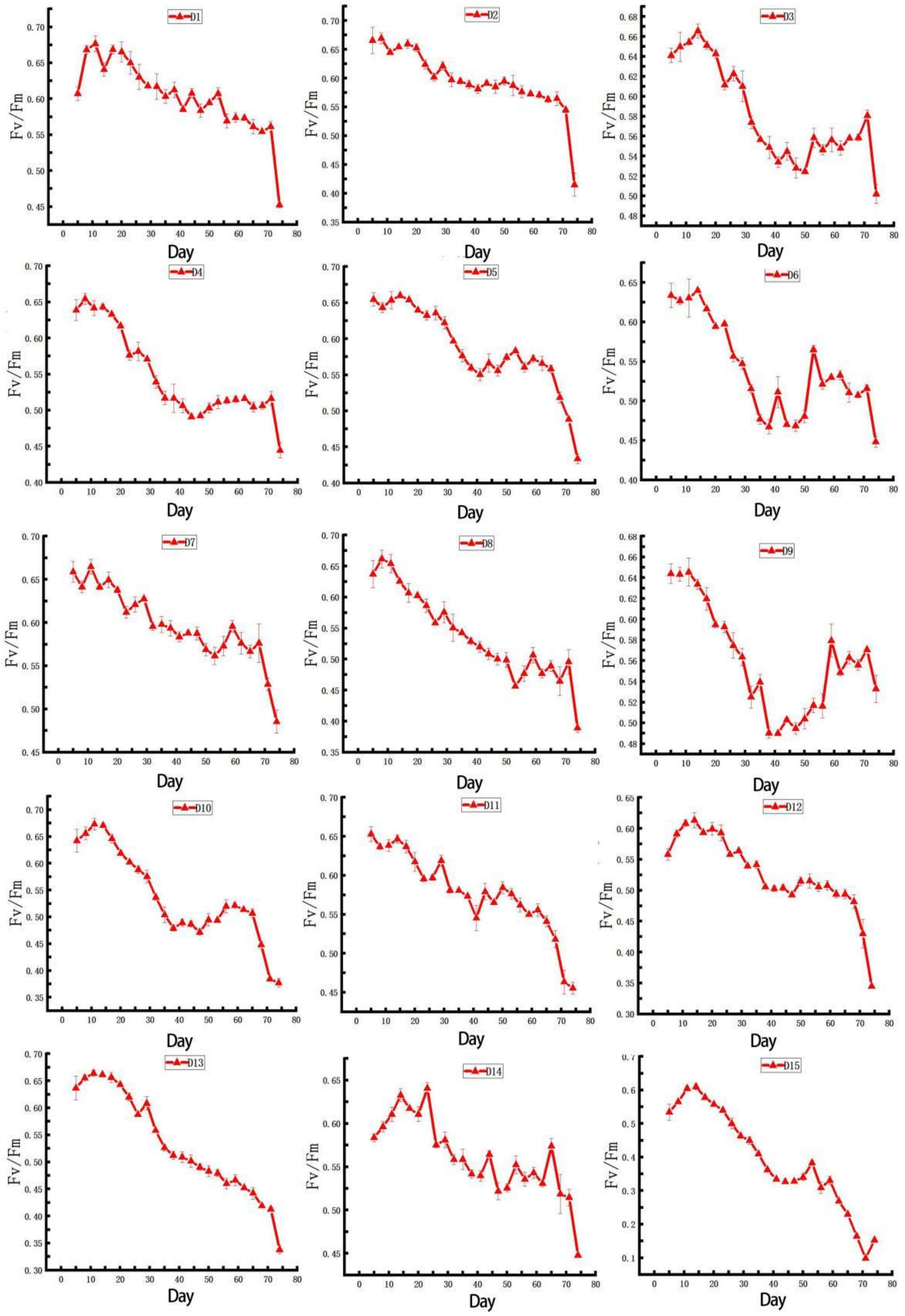

Supplementary Figure S5 Changes in the Fv/Fm ratio in the 15 *Dunaliella* species
